# Facilitating the Adoption and Evolution of Digital Technologies Through Re-conceptualization

**DOI:** 10.3389/fsurg.2022.840595

**Published:** 2022-02-22

**Authors:** Nicholas Pari Tekkis, Rebecca Richmond-Smith, Gianluca Pellino, Christos Kontovounisios

**Affiliations:** ^1^School of Clinical Medicine, University of Cambridge, Cambridge, United Kingdom; ^2^School of the Biological Sciences, University of Cambridge, Cambridge, United Kingdom; ^3^Department of Advanced Medical and Surgical Sciences, Università degli Studi della Campania “Luigi Vanvitelli”, Naples, Italy; ^4^Colorectal Surgery, Vall d'Hebron University Hospital, Barcelona, Spain; ^5^Department of Colorectal Surgery, Chelsea and Westminster Hospital, London, United Kingdom; ^6^Department of Colorectal Surgery, Royal Marsden Hospital, London, United Kingdom; ^7^Department of Surgery and Cancer, Imperial College, London, United Kingdom

**Keywords:** 3D printing, imaging, innovation, healthcare system, layered modular architecture

## Abstract

**Background:**

The NHS has been making steps toward greater efficiency and cutting costs to maintain quality of care despite constraints, but without innovation the NHS will not be able to meet its increasing financial demands. The purpose of this article is to analyse a single potentially transformative technology's path of adoption in the NHS [3D printing (3DP)].

**Methods:**

Analysis of 3DP and its current value propositions. Re-conceptualization of the technology to gain insights into these value propositions and identify the capabilities it may provide. Analysis of previous business models to identify where this value is not fully captured and development of a new business model, followed by exploration of benefits and potential limitations of this new model.

**Results:**

3D printing applications can be broadly categorized into anatomical modeling, implants, and tools. Conceptualizing 3D imaging using the layered architecture model suggests the potential of 3DP to evolve the current imaging and modeling infrastructure of the NHS, and as such should be adopted to facilitate this potential.

**Conclusion:**

3D printing is an innovation with large potential for generativity, and it is important that it is integrated at a level that could both stimulate and communicate its benefits. Re-conceptualization identified a backbone within the NHS that could facilitate it as a point of entry, and the most successful installations have been through this channel. However, progress on the frontier is currently limited by both physical and organizational boundaries, the resolution of which is paramount for the current and future success of this technology.

## Introduction

The NHS is a publicly funded organization responsible for maintaining the physical and mental wellbeing of the UK population. Thirteen percent of all jobs in the UK are in the health and care sector (1), a large fraction of which are encompassed by the NHS. In order for it to perform at such size and scope, a bureaucratic structure has been established, resulting in structural inertia and barriers to the development and implementation of new technology. This resistance is observed to a greater extent in the NHS than its international equivalents (2), likely due to being compounded by a continuous increase in financial constraint. The NHS has been making steps toward greater efficiency and cutting costs to maintain quality of care despite constraints, but without innovation the NHS will not be able to meet its increasing financial demands (2). As such, addressing its uptake of digital technology is of paramount importance to stimulate innovation and ensure its continued survival.

We analyse a single potentially transformative technology's path of adoption in the NHS [3D printing (3DP)]. Following an outline of the current value propositions, we present a re-conceptualized view of 3DP to gain insights into these value propositions and identify the capabilities it may provide. Following this we analyse previous business models to identify where this value is not fully captured. Finally, we present and explore a new business model to identify its benefits and potential limitations.

### 3D Printing in Healthcare, the Point of Entry

**Figure 1:**
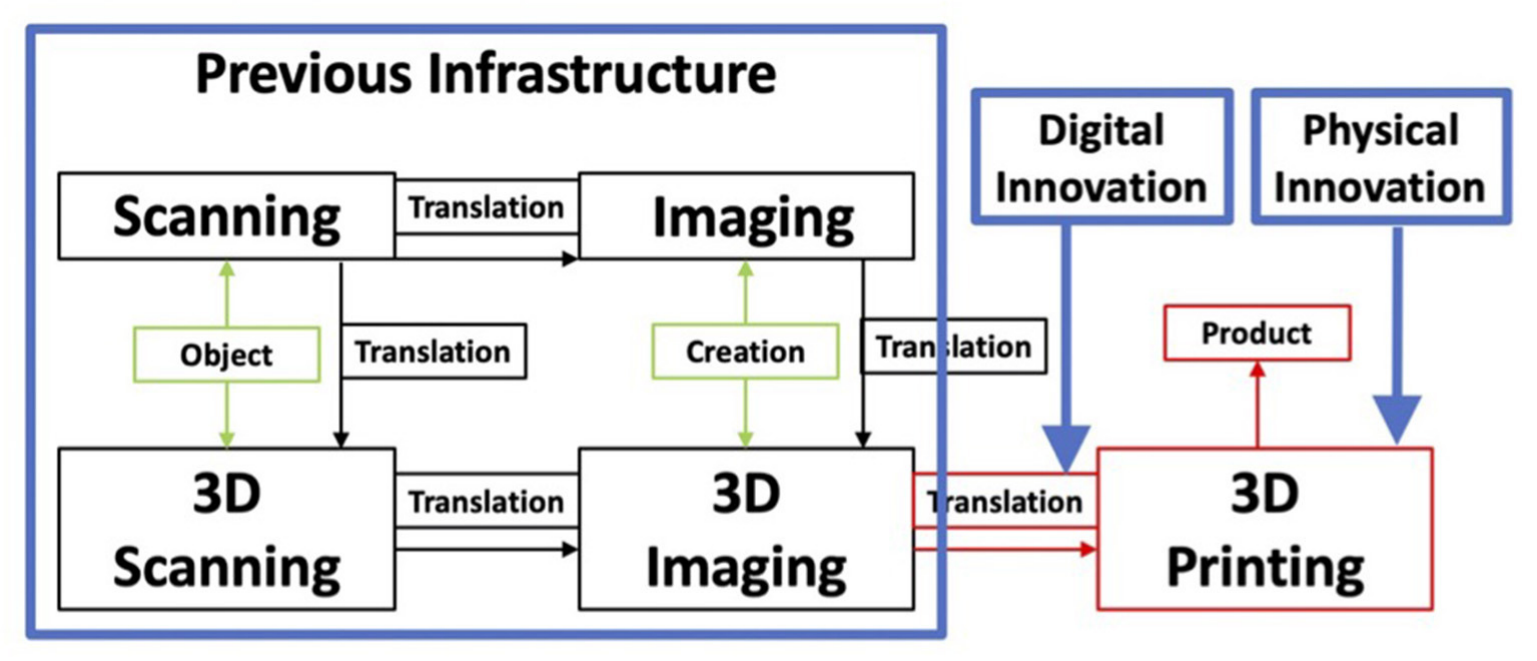
3D printing as development of its predecessors, and segmentation according to observations by Polykarpou et al. (4).

3D printing, a type of additive manufacturing, is the process of translating computer aided design models to produce 3D objects through the addition of material layer-by-layer (3). This definition highlights the duality of 3DP as both a digital and physical innovation, being the software that translates 3D models into commands for the printing apparatus and as the apparatus itself, with Polykarpou et al. (4) attributing this to Jones and Rose's (5) bridging of the digital and physical domains. As such, a more expanded view of 3DP (Figure 1) is a process that derives from a sequence of prior innovations within the digital domain, namely scanning and imaging.

In the context of medicine, scanning and imaging have fully penetrated the NHS, with extensive use of X-rays, ultrasound scans, and magnetic resonance imaging amongst others to create digital models of patients. Bailey et al. (6) highlights the benefits of virtual modeling, enabling virtual teams and remote control, e.g., radiology departments diagnosing patients and advising procedure without examination, as well as simulation, such as a surgery team planning an operation in advance by interacting with the model, as well as use in educating medical students (7). Alongside the benefits come limitations. Practitioners need to be trained to interpret the scan as well as identify where it may not be an accurate representation of reality. Furthermore, they only have access to the digital information provided. Zuboff (8) highlighted the struggles of carrying out this “informated work,” an observation which despite improvements in training and modeling, remains present today. Beyond professional use, another implication is that for the patient, who likely has little skill in interpreting scans, thus contributing to the continuous ethical struggle of acquiring true patient consent for procedures and prescriptions. 3D printing has been introduced to healthcare on the foundation of 3D imaging in part as a means of reducing the limitations of virtual models as well as to introduce new capabilities.

## Value Propositions of 3D Printing

Despite an enormous variety of current applications for 3DP, most can be categorized into three groups: anatomical modeling, implants and tools. The proposed benefits of all these vary, however are encompassed by four main categories: clinical application, patient orientation, education, and logistical improvement.

3D Modeling has been introduced as a means to counteract the limitations of digitized work whilst preserving its benefits. The printing of previously digitized models minimizes the degree of “informated work” required, aiding doctors in planning complex surgery (9), whilst increasing the range of remote planning. The proposed benefit is a more efficient use of operating time with cost reduction implications (10, 11). The same principle has been applied to educating patients, aiding in obtaining informed consent (9), as well as in clinical training. However, despite a vast array of articles detailing the surgical application of these benefits to their own niches (12), few have focused on communicating their financial value.

3D printing implants partly offer value through similar means. The development of patient specific surgical guides for implants in maxillofacial surgery have been shown to reduce operation times, with international data suggesting 33% reductions and £1,500 equivalent savings per operation (13). The value propositions of the implants themselves are mostly linked to aesthetic functionality, longevity and simplicity in procedure. Whilst the latter two can be linked to financial benefit through costs of replacements and errors, the tangible value of marginally improved aesthetic outcomes to a hospital is less direct. Social factors influence individual's decisions under bounded rationality (14), exemplified by IT investments improving hospital reputation through media attention (15), which in turn carries benefit in the form of referral, opportunities, funding, etc. Along similar lines, patient orientated products such as implants and 3D models for communication serve to improve the reputational value of the hospital that adopts them.

Newer implants explore the use of a 3D-printed mesh with cell-culture injection, as well as the direct printing of cell layers in the form of “bio-inks.” These have been used to make patient specific tissues such as skin (16), larger tissues such as knee menisci (17) and, though still in its infancy, organ printing such as ovaries. If clinical viability were established however, the logistical and reputational advantage of donor waiting-list management and transportation cost-saving would be considerable.

The proposed advantages of tools vary with the level of integration. At the procedural level, custom tools could enable better surgical outcomes, malfunction reduction, and cost savings in atypical anatomical situations e.g., laparoscopic trocars for children (18).

On the hospital level, there is an opportunity for hospital equipment design, customization, and optimization for various efficiency improvements (4) which could be amplified on an NHS scale with plaformization, amplifying innovation through the generativity a distributed innovation network provides (19). This maneuver has the possibility of generating a threat of vertical integration to NHS suppliers, increasing the buyer power of the NHS (20), potentially driving down the cost of externally sourced equipment.

## Discussion

### Re-conceptualization of 3D Printing

Our conceptualization of 3DP is a development of Polykarpou et al. (4), which highlighted the bridging of the physical and digital domains, but also separately emphasized the creation of a physical domain and reliance on a previous infrastructure. These three separate observations were combined to develop Figure 1.

**Figure 2:**
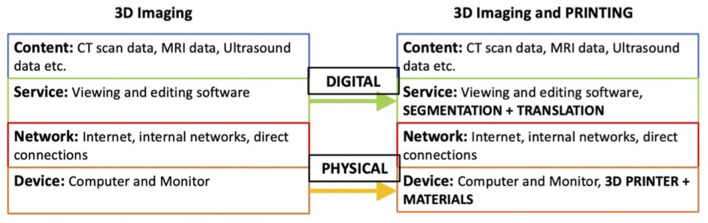
Innovation in the service and device layers of 3D Imaging's layered modular architecture.

However, analyzing the value propositions of 3DP in healthcare has shown a huge variation in applications, all stemming from an initial process innovation. This presents another key feature of 3DP, its ability to facilitate generativity, which may not be emphasized enough in previous conceptualizations. The origin of this generativity can be explained by integrating 3DP with its precursor (Figure 2).

Conceptualizing 3D imaging using the layered architecture model suggests a large degree of modularity through independent layer development. Layered modular architectures possess the intrinsic capability of stimulating generativity (21), evidenced by the plethora of applications of 3D imaging through innovations in all layers. If 3DP is conceptualized as an innovation in the service and device layers of 3D Imaging's architecture, then it can be conceptualized as a product of the architecture's modularity, and so be subject to the same modularity, thus explaining the origin of the generativity observed so far. This conceptualization shows the potential of 3DP to evolve the current imaging and modeling infrastructure of the NHS, and as such should be adopted to facilitate this potential. However, this is not fully appreciated in previous adoption models.

### Traditional Model of Adoption and Diffusion

Gartner's Hype cycle for Healthcare Providers, 2018 (22) segments 3DP into the products of its generativity. What this suggests is that 3DP is not viewed as a singular entity to be adopted but rather as a collection of separate innovations to be chosen and developed independently of each other.

The structure of the NHS encourages further segmentation, with the categories above further categorized according to niches. This is due to the NHS being highly decentralized with control over hospital funding in individual areas largely conducted by 135 Clinical Commissioning Groups (CCGs). The variation in demography across the UK results in a heterogeneity of need for different innovations between and within CCGs, encouraging innovation to be managed in niches where it is most needed. In theory, this makes adoption of technology easier, and has enabled many improvements since its introduction in 2012, an example being improved mental health care^1^. However, the large degree of organizational complexity that decentralization has brought (23) has increased the number of barriers through which knowledge would need to be exchanged, as well as amplifying the “dysfunctions” in knowledge communication across these barriers. A particular disruption of relevance is “audience learning” (24), highlighted practically as the establishment of a CCG “fortress mentality” (25), where providers prioritize their own area pressures over collaborating with other providers for greater goals, reducing the diffusion of knowledge, and by extension, innovation.

For 3DP in particular, a hospital identifies its individual needs and is provided 3DP for use in a niche that needs addressing, with a simultaneous clinical study for efficacy. However, whilst evidence of efficacy is considerable, the overall 3DP-process is often slow (26), limiting the number of patients it can help in a given time period. Furthermore, evidence of financial benefit to hospitals is scarce, and with a lack of use in surrounding departments to demonstrate further application and cost justification, the technology is not given the opportunity to grow due to concern over financial risk. Attempts to address the issues brought up are also slow. In 3D modeling, the bottleneck is the need to segment scans into specific sections for printing. This is currently done manually, taking up to 6 h. Solutions involving the hiring of technicians (27) and segmentation though machine learning have been proposed (28), however without sufficient financial evidence to justify a technician or sufficient past cases to serve as a source of information for learning, optimization to increase efficiency cannot occur.

Overall, the traditional approach prevents 3DP from integrating with and building on its previous infrastructure, thus failing to communicate all of its value, resulting in a decreased interdepartmental reach as well as reduced process improvement. In essence this is a case of “role constrained learning” amplified by the bureaucratic inflexibility of the NHS with regards to changing structure to facilitate evolution. This in turn has contributed to its lack of diffusion (learning under ambiguity), with much of the diffusion that does occur due to acknowledgment of the reputational value of digital innovation. Adoption for the sake of reputation however is usually superficial, thus expressing the same limitations in growth potential as the pioneers, and consequently is not developed further.

### Novel Adoption Approach

For 3DP's integration into the NHS to be successful, a business model would not only need to firstly facilitate 3DP's potential for generativity, but also present a value proposition that attends to the dysfunctions in knowledge transfer created by the structure of the NHS. These two are not mutually exclusive, as generativity is itself a value which can be communicated.

Radiology (imaging) exists interdepartmentally in many hospital settings, enabling multiple parties to benefit within the hospital. If 3DP lies on the same modular architecture, and is also integrated at such an interdepartmental level, it would be poised to evolve to benefit all departments in a similar manner. If all departments were provided with 3DP, setting up a distributed network, generativity would theoretically be maximized.

However, a successful business model creates, delivers and captures value (29), and in a hospital setting value exists on two levels, one for a subgroup of patients, the other for all patients under the hospitals care. Therefore, a medical business model has the added requirement of balancing one with the other. A 3DP installation would be costly and demand organizational shift, the consequences of which may affect patient care in other areas. As such, the marginal benefit (in terms of delivering value and stimulating generativity) of adding a printer must be compared to its cost. Interdepartmentalization not only stimulates generativity (creating value), but also increases the size of the patient subgroup (delivering more value), thus enabling the perceived marginal benefit of installing a small number of 3DP's to rise above their cost. Perceived cost to hospitals has decreased further from a more evolved study of 3DP's benefits, with much change in the tone of systematic reviews from Diment et al. (12) showing an uncertainty of wider application due to lack of non-anecdotal evidence, to Emile and Wexner (30) amongst others clarifying such uncertainty. Whilst this still may not communicate much financial value, based on reports from medical professionals, it communicates clinical efficacy to surrounding medical professionals, reducing “role constrained learning” and decreasing the projected costs of organizational resistance, as well as improving hospital reputation.

However, despite the changes in perceived benefit from the re-conceptualization and perceived cost from research, hospitals can often only justify the purchase of a few printers, and thus the service installed, whilst interdepartmental, is centralized, a compromise that maximizes generativity whilst minimizing cost. This is evidenced by Cambridge University Hospitals' (CUH) successful centralized 3DP service (27). Cambridge University Hospital takes the reunification of 3DP further, constructing 3D models, tools, and implants interdepartmentally, further increasing potential generativity and the size of the patient subgroup.

### Limitations of a Centralized 3D Printing Department

3D Printing requires the entry of new staff, materials and devices whilst also integrating with hospital infrastructure. It also requires skills independent of other roles within healthcare, such as an understanding the 3DP process, segmentation, and materials (27). Most 3DP hubs hire specialized technicians to fill these knowledge demands, creating differences in knowledge (explicit and tacit) between technicians and hospital staff. This creates a new boundary of knowledge and dependence, increasing the complexity of technician interactions (31) and potentially limiting productivity if this complexity cannot be overcome. With centralized, interdepartmental 3DP services these boundaries exist between all the departments spanned, amplifying complexity further. Barrett et al. (32) highlights how even a three-way interaction involving technicians for new hospital equipment can create damaging relationships, through neglect and strain. There is direct 3DP evidence of this in Polykarpou et al. (4) where failure to communicate across the pragmatic boundaries (33) between the new technicians and the incumbent engineers resulted in neglect toward the engineers and strain in the relationship between the two, ultimately contributing to a halt of the expansion of the 3DP department into workshop space under protest of the engineers.

3D printing's properties as a physical innovation adds another layer of complexity to its adoption. This is demonstrated most obviously through an interdepartmental positioning to stimulate generativity, but also logistically with the identification of areas to be repurposed for its implementation. Without sufficient attention to the relative importance of place to the relevant parties involved, growth may be limited (4).

Resolving organizational issues as they arise is a challenge, however there are a few methods to aid in resolution. Firstly, another contributor to complexity at boundary relations is novelty. If 3DP is conceptualized and integrated in a similar manner to its predecessor—radiology, then the novelty of the interaction may be reduced. However, there is a clear difference in the skills required to carry out the two functions, and as 3DP grows it will need to interact with departments previously alien to radiology or with renewed importance, such as materials procurement and surgical teams. Novelty is therefore still expected. Another potential means of resolving barriers is with boundary spanners. Given the context of the NHS and the requirement of boundary spanners to be knowledgeable and respected by both communities (34), prime targets would be the passionate physicians who attempted to integrate 3DP in the traditional approach, displaying sufficient knowledge in both peripheries. Alternatively, an increased focus on educating young doctors of the benefits of 3DP may create boundary spanners for the future. As 3DP becomes increasingly relevant with new discoveries and improvements such as the advent of organ printing, overcoming these organizational boundaries will be crucial to their implementation and effect. Should they be addressed and solved at a time of relative simplicity, the core infrastructure of the NHS will be more accommodating of the discoveries of the future.

## Conclusion

We have analyzed a case of technological implementation in the NHS where growth was limited by the channel of introduction. 3D printing is an example of an innovation with large potential for generativity, and it was important that it was integrated at a level that could both stimulate and communicate its benefits. Re-conceptualization identified a backbone within the NHS that could facilitate it as a point of entry, and the most successful installations have been through this channel. However, progress on the frontier is currently limited by both physical and organizational boundaries, the resolution of which is paramount for the current and future success of the innovation.

## Data Availability Statement

The original contributions presented in the study are included in the article/supplementary material, further inquiries can be directed to the corresponding author/s.

## Author Contributions

NT: conception, design of work, review, analysis, interpretation of findings, drafting, and revision of manuscript. RR-S: conception of work, interpretation of findings, drafting, and revision of manuscript. GP and CK: interpretation of finding and revision of manuscript. All authors have provided approval for publication and agree to be accountable for all aspects of the work.

## Conflict of Interest

The authors declare that the research was conducted in the absence of any commercial or financial relationships that could be construed as a potential conflict of interest.

## Publisher's Note

All claims expressed in this article are solely those of the authors and do not necessarily represent those of their affiliated organizations, or those of the publisher, the editors and the reviewers. Any product that may be evaluated in this article, or claim that may be made by its manufacturer, is not guaranteed or endorsed by the publisher.

## Footnotes

^1^Available online at: http://445oon4dhpii7gjvs2jih81q.wpengine.netdna-cdn.com/wp-content/uploads/2016/09/NHSCC_Support-from-the-start_final.pdf (accessed August 03, 2019).
